# Medialisation laryngoplasty with bone wax in a sheep model

**DOI:** 10.1017/S0022215122001086

**Published:** 2023-01

**Authors:** Y Nachalon, D J Cates, N Nativ-Zeltzer, P C Belafsky

**Affiliations:** 1 Department of Otolaryngology – Head and Neck Surgery, University of California, Davis, Sacramento, USA; 2 Sackler School of Medicine, Tel Aviv University, Tel Aviv, Israel

**Keywords:** Dysphonia, Voice, Larynx, Vocal Cord Paralysis, Paraffin, Laryngoplasty

## Abstract

**Objective:**

To evaluate the safety and biocompatibility of bone wax as an implant material for medialisation laryngoplasty in a large animal model.

**Methods:**

Three Dorper-cross ewes underwent type I thyroplasty of the right vocal fold with bone wax. The animals were monitored for four weeks for general wellbeing. The animals were euthanised and the larynges harvested. Histological evaluation was performed to assess for adverse tissue reaction and biocompatibility.

**Results:**

The mean (± standard deviation) amount of bone wax implanted was 0.49 g (± 0.12 g). No adverse events were reported. *Ex vivo* vibration was present on high-speed imaging for all medialised vocal folds. Histology demonstrated implanted paraffin embedded within the thyroarytenoid muscle with no evidence of resorption, a minimal inflammatory infiltrate, and a thin fibrotic capsule.

**Conclusion:**

The results of this investigation suggest that bone wax may be a safe and efficacious implant material for medialisation laryngoplasty. Further studies are necessary to assess its long-term safety and efficacy.

## Introduction

Glottic insufficiency can result in dysphonia, dyspnoea, dysphagia, an increased risk of aspiration and death from pneumonia.^1–3^ Therapeutic options can vary from voice therapy to vocal fold medialisation, achieved either through injection augmentation or laryngeal framework surgery such as medialisation laryngoplasty.^4^

Medialisation laryngoplasty aims to shift the vocal fold to the midline, thus enabling glottic closure, to assist with phonation and the Valsalva manoeuvre. The two most common implant materials utilised for medialisation laryngoplasty are Silastic® and polytetrafluoroethylene (Gore-Tex). Both have advantages and limitations, and neither are perfect (Table 1).

**Table 1.** Advantages and disadvantages of available implant materials

Parameter	Silastic	Gore-Tex
Material	Silicone	Polytetrafluoroethylene
Biocompatibility?	Yes	Yes
Handling	Time-consuming to carve	Simpler handling
Adjustments	Removing, re-shaping & re-positioning implant	Adding & re-positioning a layer
Procedure time	Longer	Shorter
Learning curve	Steep	Shallow

Paraffin in the form of bone wax (Ethicon, Somerville, New Jersey, USA) is permanent, malleable, biocompatible, readily available and inexpensive. This investigation aimed to evaluate the safety and biocompatibility of bone wax as an implant material for medialisation laryngoplasty in a large animal model.

## Materials and methods

The study was approved by the Institutional Animal Care and Use Committee. Three Dorper-cross ewes aged two years underwent type I thyroplasty to the right vocal fold. The ewes were monitored for four weeks for weight, general wellbeing and respiration, and were then euthanised. The larynx was harvested and *ex vivo* phonatory recordings were taken. For each larynx, the vocal folds were dissected from the anterior commissure to the vocal process, and laterally to the thyroid cartilage, and sent for histological examination. The non-medialised vocal fold was used as a control.

### Procedure in detail

The procedure was conducted under general anaesthesia. All ewes were induced with intravenous (IV) 4 mg/kg propofol and 5 mg/kg ketamine. Intubation was carried out with a size 9.0 endotracheal tube. Anaesthesia was maintained with 1–4 per cent isofluorane.

**Fig. 1.**
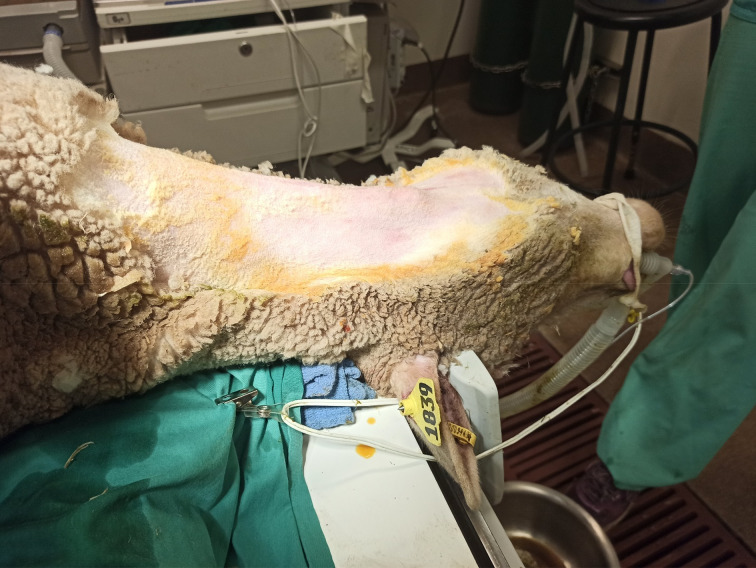
Ewe intubated under general anaesthesia. Laryngeal prominence is shown, indicating the level of the vocal folds and the height of the incision.

**Fig. 2.**
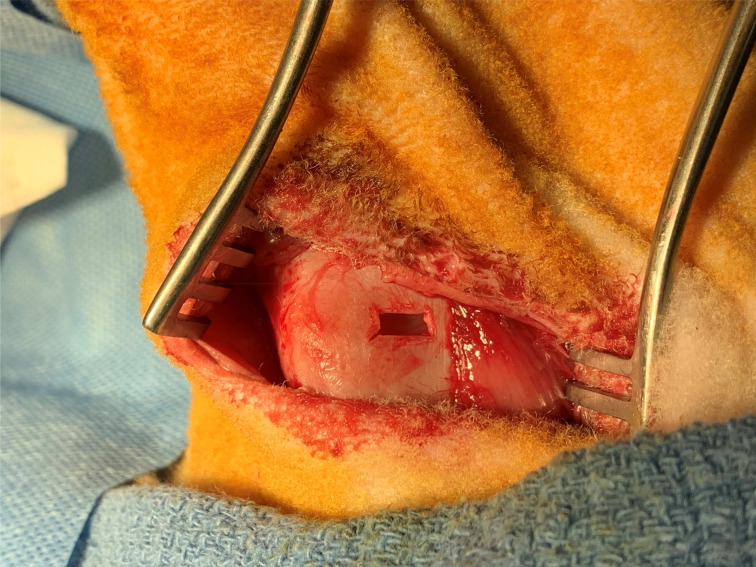
Cartilage window through the right thyroid ala. The window size was 4 × 8 mm, positioned 6 mm from the inferior border of thyroid cartilage and 6 mm lateral to the laryngeal prominence.

**Fig. 3.**
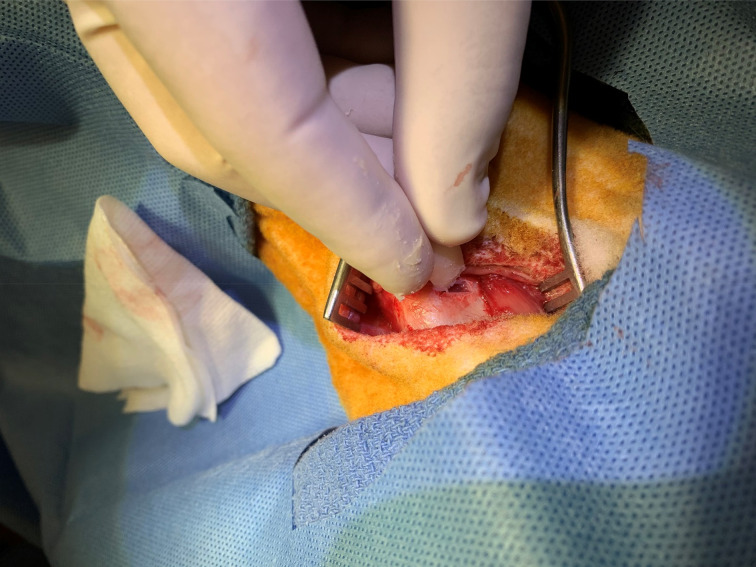
The bone wax implant material was advanced through the cartilage window.

**Fig. 4.**
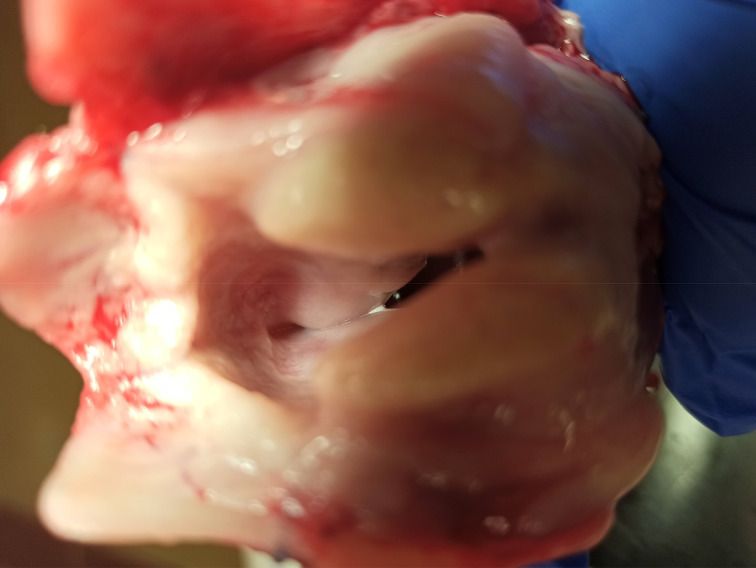
*Ex vivo* larynx with medialised right vocal fold.

The sheep were placed on the operating table in the supine position. A horizontal skin incision was made at the level of thyroid cartilage, and subplatysmal flaps were elevated to expose the larynx and strap muscles. A dissection was performed along the midline and strap muscles were retracted off the thyroid cartilage. A perichondrial flap was elevated, and a window through the thyroid cartilage lamina was created at the level of vocal folds. The implant material was advanced through the cartilage window under direct vision of the vocal fold with endoscopy to ensure proper medialisation. Perichondrial flaps were sutured with size 5-0 Prolene® sutures and the incision was closed in layers (Figures 1–4).

### Phonatory recording

Four weeks after the procedure, the ewes were euthanised with IV 100 mg/kg sodium pentobarbital. The ewes’ larynges were freshly harvested, and a phonatory recording was performed *ex vivo*.

**Fig. 5.**
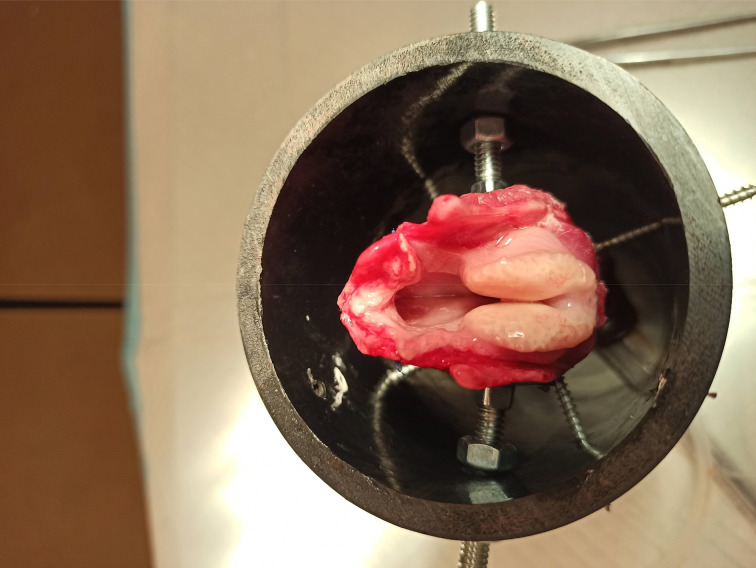
*Ex vivo* larynx mounted on a polyvinyl chloride pipe. Vocal folds are approximated medially, with pressure applied to the thyroid ala. A continuous positive airway pressure machine blows air at 20 cmH_2_O retrograde from the distal trachea towards the glottis.

The larynges were positioned in a polyvinyl chloride pipe and held with four screws at the cricoid level (Figure 5). The tissue above the vocal folds was trimmed to expose the vocal folds, and mucosa was sutured to the thyroid ala and petiole with a size 4-0 Prolene suture. A size 9.0 endotracheal tube connected to an XT Fit continuous positive airway pressure machine (Apex Medical, New Taipei City, Taiwan) was positioned retrograde from the distal end of the trachea towards the subglottis to supply airflow for phonation, at a setting of 20 cmH_2_O. Two screws on each side of the thyroid ala were advanced to apply pressure and approximate the vocal folds to a phonatory position.

Phonation was recorded using an NTG-2 directional condenser microphone (Røde Microphones, Sydney, Australia) positioned 15 cm above the vocal fold in a 45-degree angle. The microphone was connected to an AudioBox USB 96 audio interface (PreSonus, Baton Rouge, Louisiana, USA) and a laptop. Fundamental frequency was analysed using Praat acoustic signal analysis software, version 6.1.38 (Institute of Phonetic Sciences, University of Amsterdam, Netherlands). Subglottic pressure was recorded using a BE 8000 manometer (Inhealth Technologies, Carpinteria, California, USA) attached to the distal end of the endotracheal tube. Vocal fold vibration was recorded using a GoPro Hero 8 action camera (GoPro, San Mateo, California, USA) at a rate of 240 frames per second.

### Histological evaluation

After the *ex vivo* experiment, each vocal fold of the larynges was fixed in 10 per cent buffered formalin. After fixation, the tissue was embedded in paraffin. Each vocal fold was dissected in a longitudinal orientation, and a 5 μm thick section was prepared, and stained with haematoxylin and eosin.

## Results

Three ewes underwent vocal fold medialisation. All had the right vocal fold medialised with a mean (± standard deviation (SD)) of 0.49 g (± 0.12 g) bone wax implanted in the paraglottic space. All animals recovered without adverse events from the procedure. No animals displayed evidence of dyspnoea. Mean (± SD) weight gain during the study period was 13 kg (± 11 kg) and no animal lost weight during the study period.

*Ex vivo* vibration was present on high-speed imaging for all medialised vocal folds. Mean (± SD) fundamental frequency was 98.3 Hz (± 6.7 Hz) and mean (± SD) subglottal pressure was 18 cmH_2_O (± 1 cmH_2_O). The normative oscillation frequency for sheep larynges (± SD) has previously been reported as 114 Hz (± 33 Hz).^5^

**Fig. 6.**
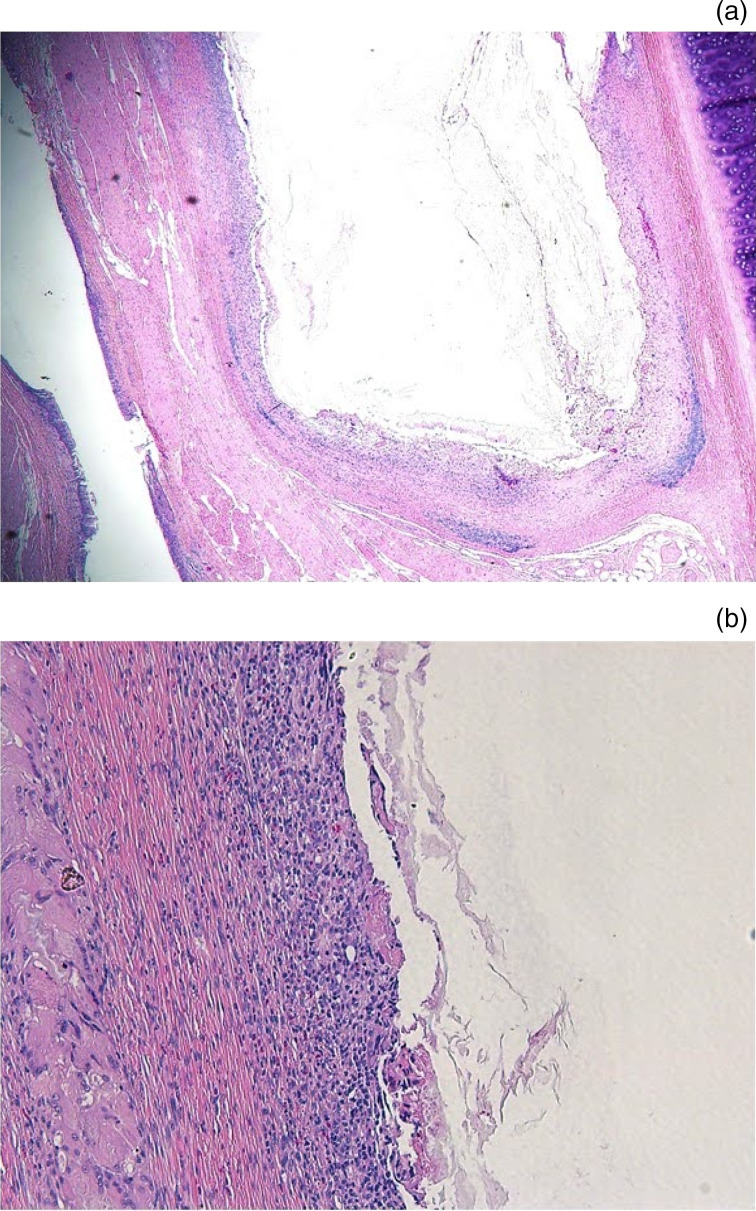
Histological evaluation images. (a) The large central clear space contains small amounts of wispy amphophilic acellular material (bone wax), surrounded by a compressed band of fibrous connective tissue with high numbers of inflammatory infiltrates. (b) The tissue around the acellular amphophilic material (bone wax) is infiltrated by high numbers of macrophages and lymphocytes, and surrounded by a rim of fibrous connective tissue and an aggregate of multinucleated giant cells. (H&E; a. ×10; b. ×40)

Histology demonstrated implanted paraffin embedded within the thyroarytenoid muscle with no evidence of resorption, a minimal inflammatory infiltrate, and a thin fibrotic capsule (Figure 6).

## Discussion

Medialisation laryngoplasty has gained popularity since it was first described by Isshiki *et al*. in 1974.^6^ The procedure has been shown to improve dysphonia and dysphagia due to glottic insufficiency. Medialisation laryngoplasty is considered relatively safe, although complications such as infection and excursion may occur.^7^

The two most common implant materials utilised for medialisation laryngoplasty are Silastic and Gore-Tex. Both have advantages and limitations. Silastic is silicone based, and can be carved and individually tailored to the patient.^8,9^ Although Silastic is relatively biocompatible, it is expensive and time-consuming to carve. This prolongs procedure time and necessitates a learning curve to achieve optimal results. Given these issues, the search for a different material continued.

The use of Gore-Tex was initially described by McCulloch and Hoffman in 1998, and has since been popularised because of its simplicity.^10^ A survey performed by Young *et al*. in 2010 demonstrated a shift from the utilisation of Silastic and an increase in the use of Gore-Tex in medialisation laryngoplasty.^11^ Suehiro *et al*. compared vocal outcomes of patients who underwent medialisation laryngoplasty with Gore-Tex and Silastic implant materials.^12^ Individuals who were medialised with Gore-Tex had greater improvement in noise-to-harmonic ratio, suggesting improved efficacy. The surgery duration was also shorter among individuals medialised with Gore-Tex. Gore-Tex, however, has its own disadvantages. Each Gore-Tex implant costs several hundred US dollars, and medialisation with the Gore-Tex strip limits the surgeon's ability to direct the pressure vector, as is possible with an individually carved Silastic implant.

We have been using bone wax to seal the thyroplasty window in Gore-Tex medialisation procedures for years. We have found that the wax readily moulds to the shape of the window and has been an easy and efficient way to keep the Gore-Tex from extruding. During a recent revision Gore-Tex thyroplasty performed three years after the initial surgery, we found the bone wax intact within the window, with no gross evidence of degradation or inflammation. It was easily removed and the medialisation was revised without difficulty.

Because of the limitations of existing materials, and our experience of bone wax used to seal thyroplasty windows as being safe and efficacious, we evaluated the use of bone wax as a primary implant material for thyroplasty.

Several reports in the 1920s described paraffin injection to the vocal folds.^13^ These reports raised concerns among clinicians regarding embolisation of the paraffin and paraffinoma. Although the reported rates of paraffinoma were very low, these concerns may partially explain the diminished use of paraffin as a vocal fold injectable.

Data from the current investigation suggest that the wax is easily advanced through the thyroid cartilage window and its use resulted in good medialisation of the vocal fold. No adverse reaction was documented during or after the procedure. Examination of the larynges *ex vivo* four weeks after the procedure revealed that the bone wax remained present, with no resorption and only mild local tissue reaction, similar to other approved implant materials. On simulated phonation, vibration was demonstrated, without evidence of vocal fold stiffness.

- The two most common implant materials utilised for medialisation laryngoplasty are Silastic and Gore-Tex
- Both have advantages and limitations, and neither are perfect
- Bone wax is permanent, malleable, biocompatible, readily available and inexpensive
- Type I thyroplasty performed in three ewes using bone wax was uneventful
- *Ex vivo* vibration was present, and histology demonstrated the implant embedded within the thyroarytenoid muscle, with no evidence of resorption

The use of bone wax as a thyroplasty implant material has some potential advantages. It is permanent, malleable, biocompatible, readily available, and inexpensive compared to either Gore-Tex or Silastic. The data from this investigation suggest that its use in the paraglottic space is safe in the short term (e.g. four weeks). Further research is required to evaluate its long-term safety and potential efficacy.

This study is not without limitations. The study sample size and follow-up time were both limited. The sheep model is an approximation of the human condition. In addition, our model of *ex vivo* vocal fold vibration is a mere surrogate for *in vivo* human strobovideolaryngoscopy. Long-term safety and efficacy cannot be confirmed from this data. Nonetheless, this study suggests that short-term (four-week) use of bone wax as a thyroplasty implant material is feasible and safe, results in a minimal inflammatory reaction, and does not limit vocal fold vibration. Further research is warranted.

## Conclusion

The results of this investigation suggest that bone wax may be a safe and efficacious implant material for medialisation laryngoplasty. Further studies are necessary to assess its long-term safety and efficacy.
